# Basic surface features of nuclear FKBPs facilitate chromatin binding

**DOI:** 10.1038/s41598-017-04194-7

**Published:** 2017-06-19

**Authors:** Andrew Leung, Francy-Pesek Jardim, Neda Savic, Yoan R. Monneau, Rodrigo González-Romero, Geoff Gudavicius, Jose M. Eirin-Lopez, Till Bartke, Cameron D. Mackereth, Juan Ausió, Christopher J. Nelson

**Affiliations:** 1 Department of Biochemistry & Microbiology, University of Victoria, Victoria, BC V8W 3P6 Canada; 2 Univ. Bordeaux, Inserm, CNRS, ARNA Laboratory, U1212, UMR 5320, Institut Européen de Chimie et Biologie, 2 rue Robert Escarpit, 33076 Pessac, France; 3 Chromatin Structure and Evolution (Chromevol) Group, Department of Biological Sciences, Florida International University, North Miami, FL USA; 4 MRC London Institute of Medical Sciences (LMS), Du Cane Road, London, W12 0NN UK; 5 Institute of Clinical Sciences (ICS), Faculty of Medicine, Imperial College London, Du Cane Road, London, W12 0NN UK

## Abstract

The nucleoplasmin family of histone chaperones is identified by a pentamer-forming domain and multiple acidic tracts that mediate histone binding and chaperone activity. Within this family, a novel domain organization was recently discovered that consists of an N-terminal nucleoplasmin-like (NPL) domain and a C-terminal FKBP peptidyl-proline isomerase domain. *Saccharomyces cerevisiae* Fpr4 is one such protein. Here we report that in addition to its known histone prolyl isomerase activities, the Fpr4 FKBP domain binds to nucleosomes and nucleosome arrays *in vitro*. This ability is mediated by a collection of basic patches that enable the enzyme to stably associate with linker DNA. The interaction of the Fpr4 FKBP with recombinant chromatin complexes condenses nucleosome arrays independently of its catalytic activity. Based on phylogenetic comparisons we propose that the chromatin binding ability of ‘basic’ FKBPs is shared amongst related orthologues present in fungi, plants, and insects. Thus, a subclass of FKBP prolyl isomerase enzymes is recruited to linker regions of chromatin.

## Introduction

The assembly and disassembly of nucleosomes is mediated by ATP-dependent and independent histone chaperones. These proteins aid in the storage, transport, deposition, and eviction of histones during transcription, as well as facilitating the replication and repair of DNA (reviewed in ref. 1). Nucleoplasmin (NPM) was the first protein shown to have this activity^2^, and it was during work on NPM that the term “chaperone” was coined^3^. *Xenopus laevis* NPM facilitates the storage of histones in advance of their deposition on sperm chromatin after fertilization^2^. A hallmark of nucleoplasmin is a protease-resistant pentameric core, which can load up to five H2A/H2B dimers or complete histone octamers, one complex per subunit^4^. While nucleoplasmin proteins do not contain additional domains, a nucleoplasmin-like (NPL) domain was recently found in several yeast, insect, and plant chromatin regulatory proteins^5^. Included in this group is the NPL-FKBP sub-family that has a unique pairing of pentamer-forming NPL and FK506-binding (FKBP) peptidyl-prolyl isomerase domains. Both of these domains modify chromatin.

**Figure 1:**
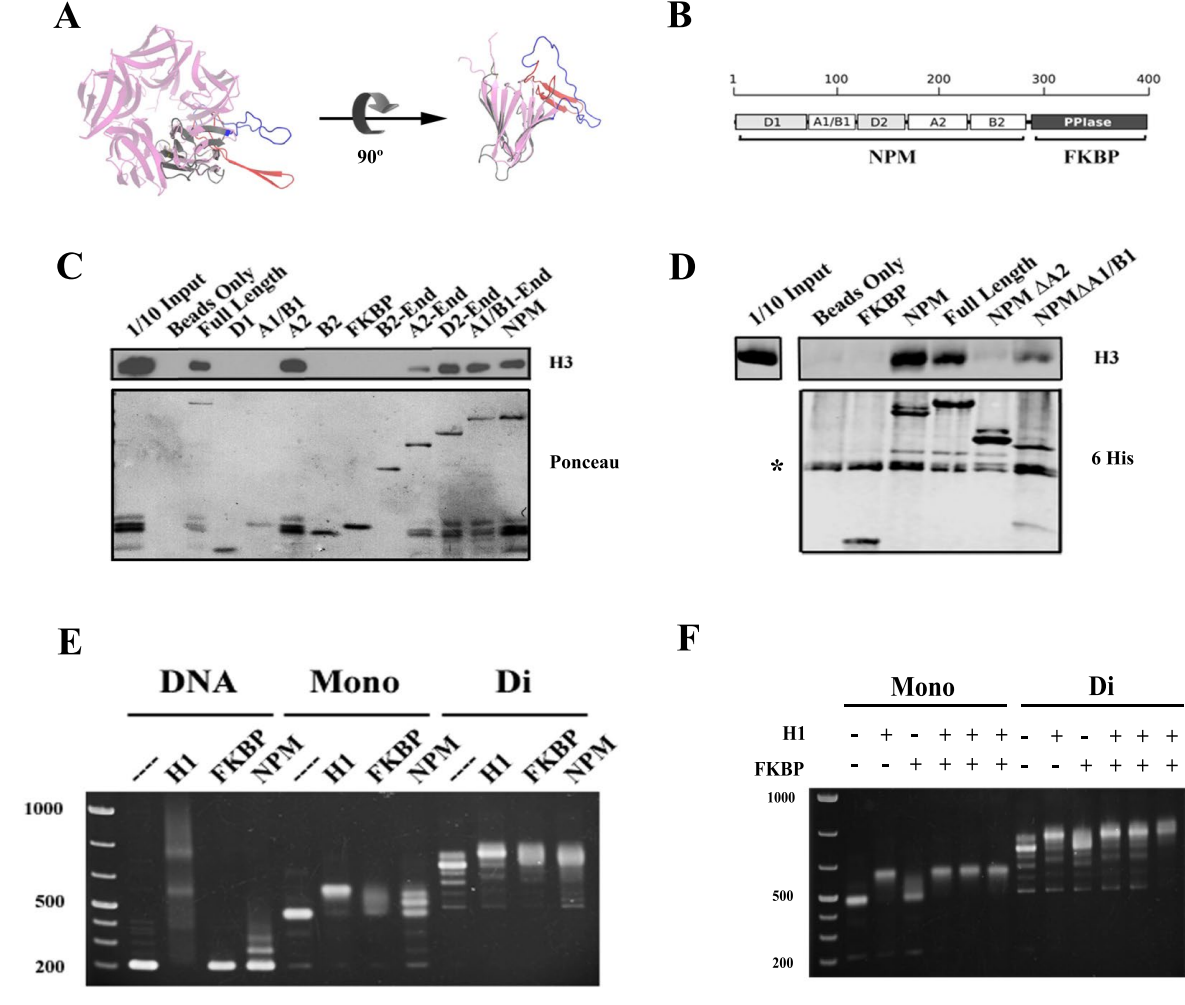
Fpr4 interacts with nucleosomes through the NPL and FKBP domains. (**A**) Predicted structure of Fpr4 NPM core domain. Fpr4 amino acids 1–169 are overlayed with crystal structure of human nucleoplasmin NPM1 (pink, PDB:1K5J). The location of the A1/B1 loop is coloured in red for the acidic portion and blue for the basic portion (amino acids 59–116), while domains D1 and D2 are coloured grey. (**B**) Domain architecture of Fpr4. Fpr4 NPL domain is comprised of a core homopentamerization motif encoded by regions D1 and D2, and is bifurcated by the A1/B1 loop. A second acidic (A2) and basic (B2) region follow before the Fpr4 FKBP prolyl isomerase domain. (**C**) The A2 region of Fpr4 is sufficient for histone binding. Histone pulldowns using calf thymus bulk histones and 6-His Fpr4 deletion constructs. Interactions were resolved by western blotting with anti-H3. Loading is shown by Ponceau staining of nitrocellulose membrane. Full-length images of this cropped blot are available in the Supplementary Information File. (**D**) The A2 region of Fpr4 is necessary for histone binding. Histone pulldowns using recombinant human histone H3 and either 6-His Fpr4 or indicated deletion mutants. Loading is shown by western blotting with anti-6-His. *Denotes non-specific band. Full-length images of this cropped blot are available in the Supplementary Information File. (**E**) The NPL and FKBP domains bind to nucleosomes. Electrophoretic mobility shift assay using either naked 601 template DNA, mononucleosomes or dinucleosomes. Recombinant histone H1, Fpr4 NL (1–280) or Fpr4 FKBP (280–392) were added to indicated substrates and binding inferred from mobility shifts visualized by agarose gel electrophoresis and ethidium bromide staining. (**F**) Histone H1 and Fpr4 FKBP do not co-occupy nucleosomes. Recombinant histone H1 was added to monucleosomes or dinucleosomes before (lanes 4, 10), after (lanes 5, 11), or simultaneously (lanes 6, 12) to the addition of Fpr4 FKBP. Gel shifts were visualized agarose gel electrophoresis and ethidium bromide staining.

The NPL domain of fly FKBP39 can be crosslinked to histones *in vitro* ^5^, and the NPL of yeast Fpr4 is located within the minimal histone binding^6^ and nucleosome chaperoning^7, 8^ region of the protein. Interestingly, as shown in Fig. 1A and B, the yeast NPL-domain is bifurcated by a 58-amino acid insertion of charged amino acids (A1/B1 loop), and is followed by a second highly-acidic region (A2). The FKBP prolyl isomerase domain of yeast Fpr4 can catalyse *cis-trans* isomerization of free peptides corresponding to histone H3 Pro16, Pro30 and Pro38 motifs^6, 9^. The proline isomer states at Pro16 and Pro38 have been implicated in crosstalk with adjacent acetylation and methylation events and these prolines are important for the expression select genes^6, 10^. While peptidyl-prolyl isomerization leads to a large structural rearrangement within a peptide, the impact of isomerization of histone tails in the context of chromatin fibres has not been examined.

Here we subjected yeast Fpr4 to a detailed *in vitro* molecular analysis to determine its binding activity to histones, nucleosomes, and nucleosome arrays. We focused our efforts on the known important sequence features of this enzyme including the NPL domain, the highly-charged regions (A1/B1 and A2) and the C-terminal FKBP domain. Using histones, mono/di nucleosomes, and 25 mer nucleosome arrays as substrates, we show that while acidic motifs within and adjacent the NPL domain are the major determinants of free histone binding, the C-terminal FKBP domain has a distinct chromatin-binding activity that is separate from its catalytic prolyl isomerase action. We identify evolutionarily-conserved basic features of the FKBP fold that enable direct binding to linker regions between nucleosomes. These basic surface patches are conserved in NPL-FKBP proteins in yeasts, insects and plants, implying an important function for this FKBP subclass. Together our results support a model where separate domains of NPL-FKBPs permit chromatin association and the chaperoning of nucleosomes.

## Results

### Acidic-tracts mediate Fpr4 interaction with histones

*Saccharomyces cerevisiae* Fpr4 was recently shown to contain a bifurcated nucleoplasmin-like (NPL) domain^5^. Amino acids 1–59 and 116–169 form the classical nucleoplasmin core but are separated by a highly-charged acidic/basic loop (A1/B1 loop) that is predicted to protrude from the nucleoplasmin fold (Fig. 1A). The NPL domain is followed by a second highly acidic region (A2; 169–226), a basic region (B2; 226–280), and the FKBP domain (280–392), (Fig. 1B). Since the A1/B1 loop and A2 region are within the minimal region of histone binding^6, 7^ we tested if these regions interact directly with histones using pulldowns with 6His-tagged deletions of Fpr4 (Fig. 1C). We find that the highly-acidic A2 region is sufficient for histone H3 interaction because the A2 region alone, or any deletion construct containing it, captures histone H3 (Fig. 1C). The A2 region is also necessary for H3 binding because Fpr4 constructs lacking the A2 region cannot stably interact with histone H3 (Fig. 1D). By contrast, the A1/B1 loop appears to play a minor role in histone interaction: it does not bind histone H3 (Fig. 1C), and Fpr4 constructs lacking this loop retain moderate H3 binding activity (Fig. 1D). Together these data demonstrate that acidic regions outside (A2) and within (A1/B1) the NPL are major and minor histone binding motifs, respectively.

### The NPL and FKBP domains mediate interaction of Fpr4 with nucleosomes

Histone chaperones, including nucleoplasmin, catalyze nucleosome deposition and removal^1^ so they must interact with both free and chromatinized histones. Given that Fpr4 can be cross-linked to chromatin *in vivo* ^6, 8^, we next sought to determine which regions of Fpr4 mediate its interaction with chromatin. To this end, we generated both mono and dinucleosomes on 197 bp 601 Widom DNA^11^ and used these complexes as substrates in electrophoretic mobility shift assays (EMSAs) with two non-overlapping regions of Fpr4: the 1–280 region that harbors all reported histone binding activity and 280–392 that comprises the FKBP prolyl-isomerase fold that does not bind free histones (Fig. 1C,D). Histone H1 was included as a positive control. As expected and previously reported^12, 13^, histone H1displays moderate binding to free DNA and strong interaction with both mono and dinucleosome complexes (Fig. 1E). By contrast, Fpr4 1–280 has a low level of DNA interaction, and a robust interaction with mono and dinucleosomes (Fig. 1E, NL). This is expected, given its direct binding to histone tails^6^. Surprisingly, although the Fpr4 FKBP does not interact with free histones (Fig. 1D) or free DNA (Fig. 1E) we observed an interaction between this FKBP and both mono and dinucleosomes, indicating preferential recognition of chromatin. As this behavior is similar to that of H1-nucleosome interactions we sought to compare H1-nucleosome and FKBP-nucleosome complexes. Specifically, we asked if H1 and the Fpr4 FKBP could simultaneously bind to nucleosomes. Regardless of the order of protein addition we find that mixtures of the FKBP domain and H1 yield H1-like EMSA supershifts and not a larger trimeric complex (Fig. 1F). These results demonstrate that histone H1 has a higher affinity for the nucleosome than the Fpr4 FKBP, and that overlapping features of the nucleosome are recognized by these proteins.

### The Fpr4 FKBP interacts with the DNA entry/exit region of nucleosomes

**Figure 2:**
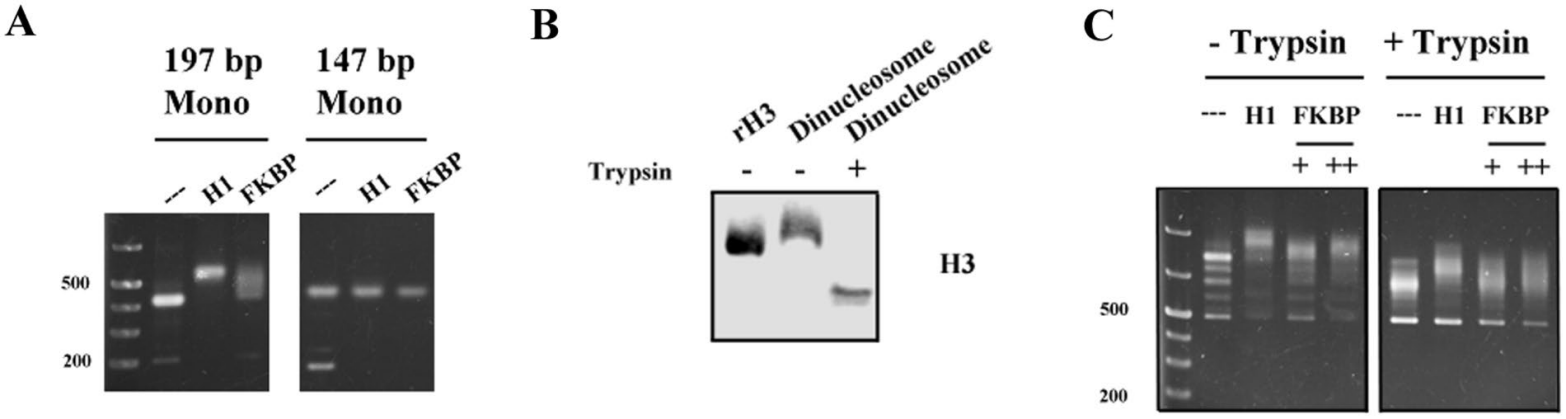
Fpr4 FKBP-nucleosome interaction requires linker DNA and histone tails. (**A**) Linker DNA is required for FKBP interaction with mononucleosomes. Nucleosomes assembled with 197 bp and 147 bp Widom 601 nucleosome positioning sequence DNA were incubated with no protein, histone H1 or the Fpr4 FKBP domain and subjected to EMSA as described in the Methods. (**B**) Anti-H3 Western blot analysis of trypsinized dinucleosomes. Recombinant human histone H3 was used as a positive control. Full-length images of this cropped blot are available in the Supplementary Information File. (**C**) FKBP interaction with nucleosomes is influenced by histone tails. Recombinant histone H1 or two concentrations of Fpr4 FKBP were mixed with intact or trypsinized dinucleosomes and visualized by agarose gel electrophoresis with ethidium bromide staining.

We next asked which features of the nucleosome are recognized by the Fpr4 FKBP domain. We first tested the requirement of linker DNA by repeating EMSA experiments with 197 bp and 147 bp 601 mononucleosomes (Fig. 2A). Like H1, the Fpr4 FKBP failed to interact with 147 bp nucleosome core particles. Thus, linker DNA mediates the FKBP-nucleosome interaction. While the FKBP domain of Fpr4 does not stably bind to histones, it does catalyze the isomerization of three prolines in the H3 tail^6, 9^. To determine if the accessible histone tails represent a second feature of the FKBP interaction surface, we removed them via limited trypsin digestion of dinucleosomes (Fig. 2B), and repeated EMSAs. As shown in Fig. 2C, while tailless dinucleosomes are still supershifted by histone H1, we note a reduction in FKBP-binding to the dinucleosomes that lack histone tails (Fig. 2D). Collectively, these results suggest that the linker DNA is required for the Fpr4 FKBP to bind to nucleosomes, while histone tails may modulate this interaction.

### The Fpr4 FKBP occupies linker regions and promotes chromatin fibre self-association independent of catalytic activity

**Figure 3:**
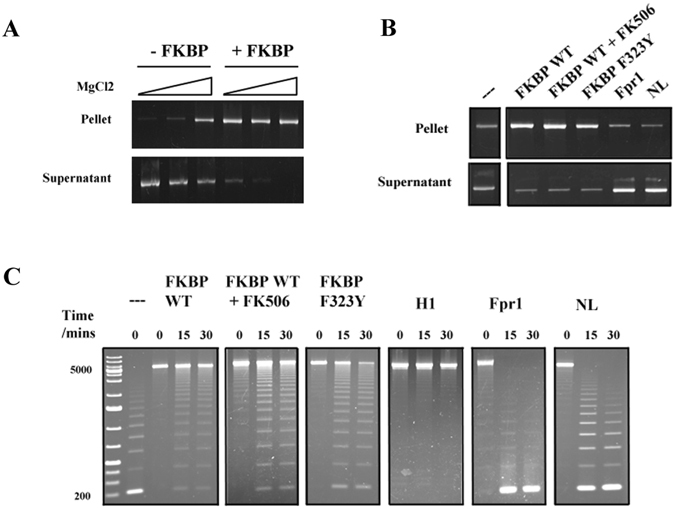
Fpr4 FKBP condenses nucleosome arrays. (**A**) The Fpr4 FKBP sensitizes nucleosome arrays to magnesium-dependent self-association. Recombinant Fpr4 FKBP was mixed with 25 mer nucleosome array in the presence of 0.25 mM, 0.5 mM and 1 mM MgCl_2_ and centrifuged. Nucleosome arrays in the supernatant and pellet fraction were visualized by agarose gel electrophoresis with ethidium bromide staining. The supernatant and pellet fractions contain extended and condensed arrays, respectively. Full-length images of this cropped gel are available in the Supplementary Information File. (**B**) Chromatin fibre self-association is unique for Fpr4 but does not require peptidyl-prolyl isomerase activity. Recombinant Fpr4 FKBP, FK506-treated Fpr4 FKBP, Fpr4 FKBP F323Y, Fpr1 and Fpr4 NL were mixed with nucleosome arrays in the presence of 0.5 mM MgCl_2_ and subjected to centrifugation to separate condensed and open fibres. Full-length images of this cropped gel are available in the Supplementary Information File. (**C**) The Fpr4 FKBP occupies linker regions in nucleosome arrays. Recombinant Fpr4 FKBP, FK506-treated Fpr4 FKBP, Fpr4 FKBP F323Y, Fpr1 and Fpr4 NL were mixed with 25 mer nucleosome array, and arrays digested with AvaI restriction enzyme for 15 and 30 minutes. DNA was purified and subjected to agarose gel electrophoresis and ethidium bromide staining. DNA from arrays with fully protected AvaI sites migrates at 5000 bp while digested arrays liberate fragments in 197 bp increments.

Histone H1 binds to linker DNA to induce chromatin folding^14^. This binding has an effect on chromatin solubility *in vitro* that is readily apparent when magnesium is applied in a dose dependent fashion, with H1-containing chromatin self-associating at lower magnesium concentrations^15^. Given our results that Fpr4 FKBP interacts with nucleosomes similarly to histone H1, we next sought to determine if the Fpr4 FKBP also influences the architecture of chromatin fibres. To test this, we generated homogeneous 25-nucleosome arrays (as we have previously described)^16, 17^. When treated with increasing magnesium concentrations this synthetic chromatin template exhibits a solubility that closely resembles that which would be expected from a dose-dependent self-association^18, 19^, with ~70% of arrays self-associating at 1mM MgCl_2_ (Fig. 3A). The pre-incubation of arrays with Fpr4 FKBP causes a dramatic decrease in array self-association at all magnesium concentrations with >90% of the chromatin shifted to the pellet fraction at the lowest (0.25 mM) magnesium concentration tested (Fig. 3A).

To determine if Fpr4 prolyl isomerase action mediates chromatin fibre self-association, we repeated these experiments with FK506-treated Fpr4 FKBP and an F323Y point mutant that is folded but lacks catalytic activity^7, 20^. In both cases, the FKBP retains the ability to condense chromatin, demonstrating that prolyl isomerase activity does not induce self-association of nucleosome arrays. Instead, these data imply that another feature of the FKBP of Fpr4 must mediate the change chromatin architecture. Notably, this feature is specific to the FKBP of Fpr4 because Fpr1, a cytosolic single-domain FKBP in yeast, could not induce pelleting of nucleosome arrays (Fig. 3B).

Finally, we used digestion at AvaI restriction enzyme sites (located in the linker DNA sequence of our arrays) as a tool to independently measure the accessibility of linker DNA in the presence and absence of the Fpr4 FKBP. In agreement with magnesium-induced self-association experiments, these assays demonstrate that arrays treated with histone H1 and Fpr4 FKBP have a distinct protected topology (Fig. 3C). By contrast, Fpr1, or the remainder of Fpr4 that contains the nucleoplasmin-like domain (NL, amino acids 1–280), have no effect on linker region accessibility. Again, the use of FK506 and a catalytically inactive F323Y mutant confirm that this effect is independent of prolyl isomerase activity. Collectively these results show that the FKBP domain of Fpr4 binds to nucleosome arrays at linker regions and that the catalytic action of the FKBP enzyme is not needed for fibre self-association *in vitro*.

### Conserved basic patches in FKBP domains mediate nucleosome binding and chromatin fibre self-association

**Figure 4:**
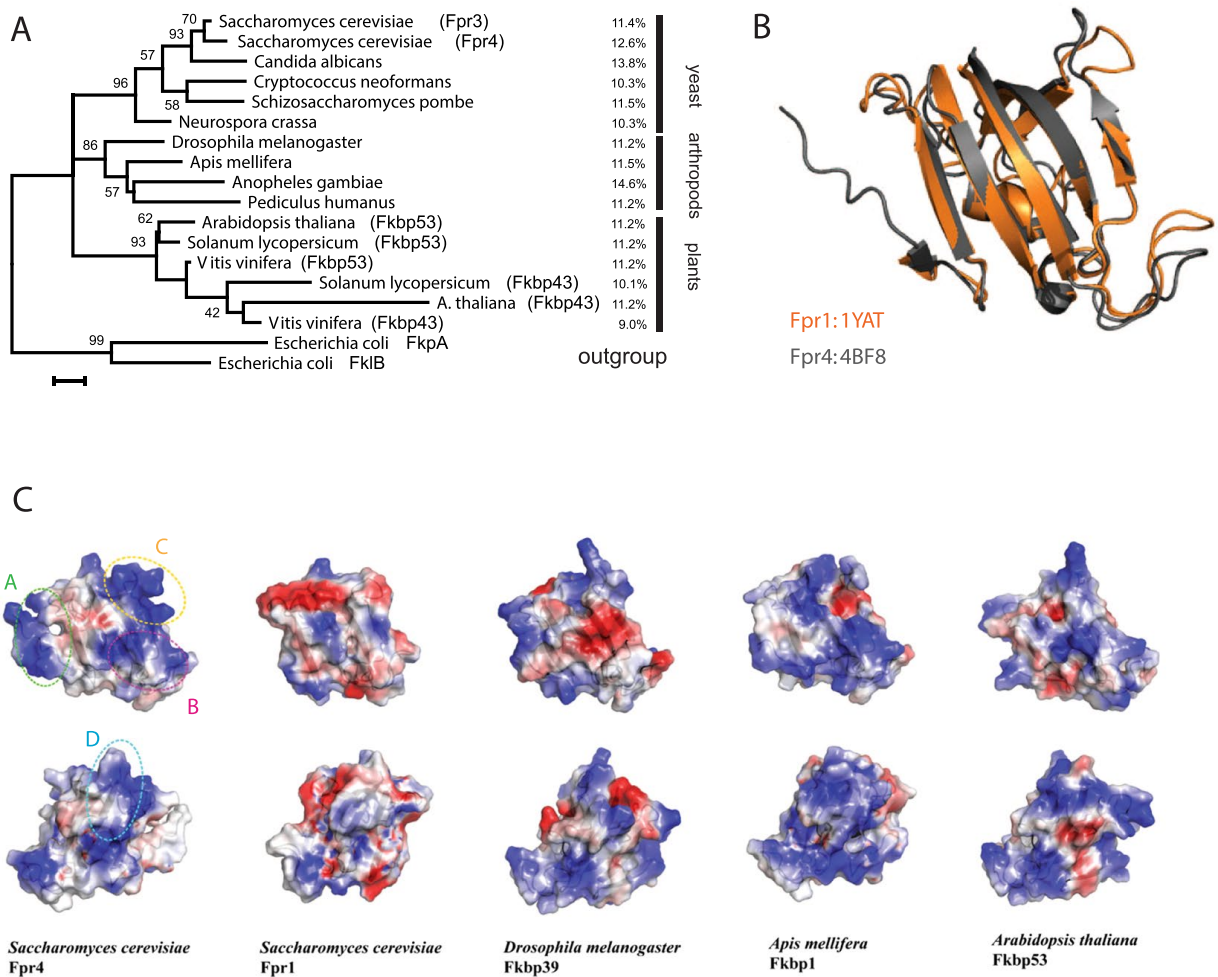
NCL-FKBPs contain conserved basic patches in their FKBP domains. (**A**) Evolutionary relationships and electrostatic characteristics of NPL-FKBP proteins in eukaryotes. Protein phylogeny describing the relationship among NPL-FKBP sequences from different phyla, including yeast, arthropods and plants. Lysine content for each sequence within each group is indicated in the right margin of the figure. The tree was reconstructed based on FKBP domains using maximum-likelihood (substitution model WAG + G + I). Values in nodes indicate bootstrap values (only shown when ≥50%). (**B**) Sequence differences between yeast Fpr1 and Fpr4 do not alter the fold of the FKBP domain. Alignments the NMR structures of 1YAT ref. 32 and 4BF8 refs 9 and 33. (**C**) Basic surface patches in NPL-FKBP catalytic domains are generally conserved. The FKBP domains from the indicated orthologous FKBP domains in flies (*Drosophila melanogaster*), honey bees (*Apis mellifera*) and plants (*Arabidopsis thaliana*) were aligned to the Fpr4 catalytic domain (PDB:4BF8) and their electrostatic surface features represented in MacPymol. Structures are rotated 180 degrees horizontally. Red = acidic side chain; Blue = basic side chain. Four basic patches (**A**–**D**) are comprised of 3–5 amino acids each, as specified in the text.

A comparison of the FKBP domains of Fpr1 and Fpr4 reveals that the later has many more basic residues and this is reflected in the isoelectric points of these proteins (pI Fpr4 FKBP = 10.2 vs pI Fpr1 = 5.7). Such basic FKBPs are not exclusive to *S. cerevisiae*. An alignment (not shown) and phylogenetic analysis of NPL-FKBPs found in yeasts, plants, and insects demonstrates that the FKBPs of these proteins are always Fpr4-like: ie. they are lysine-rich and consequently have relatively high pIs (Fig. 4A). A comparison of the NMR structures of yeast Fpr4 and Fpr1, indicates that this basic character does not alter the fold of the domain (Fig. 4B) but creates four basic patches on the surface (Fig. 4C). In general, these areas are also found in the FKBPs of the NPL-FKBP family, as shown for fruit fly (*D. melanogaster*), honey bee (*A. mellifera*), and plant (*A. thaliana*) (Fig. 4C), suggesting that this basic character is likely important for the function of NPL-FKBPs.

**Figure 5:**
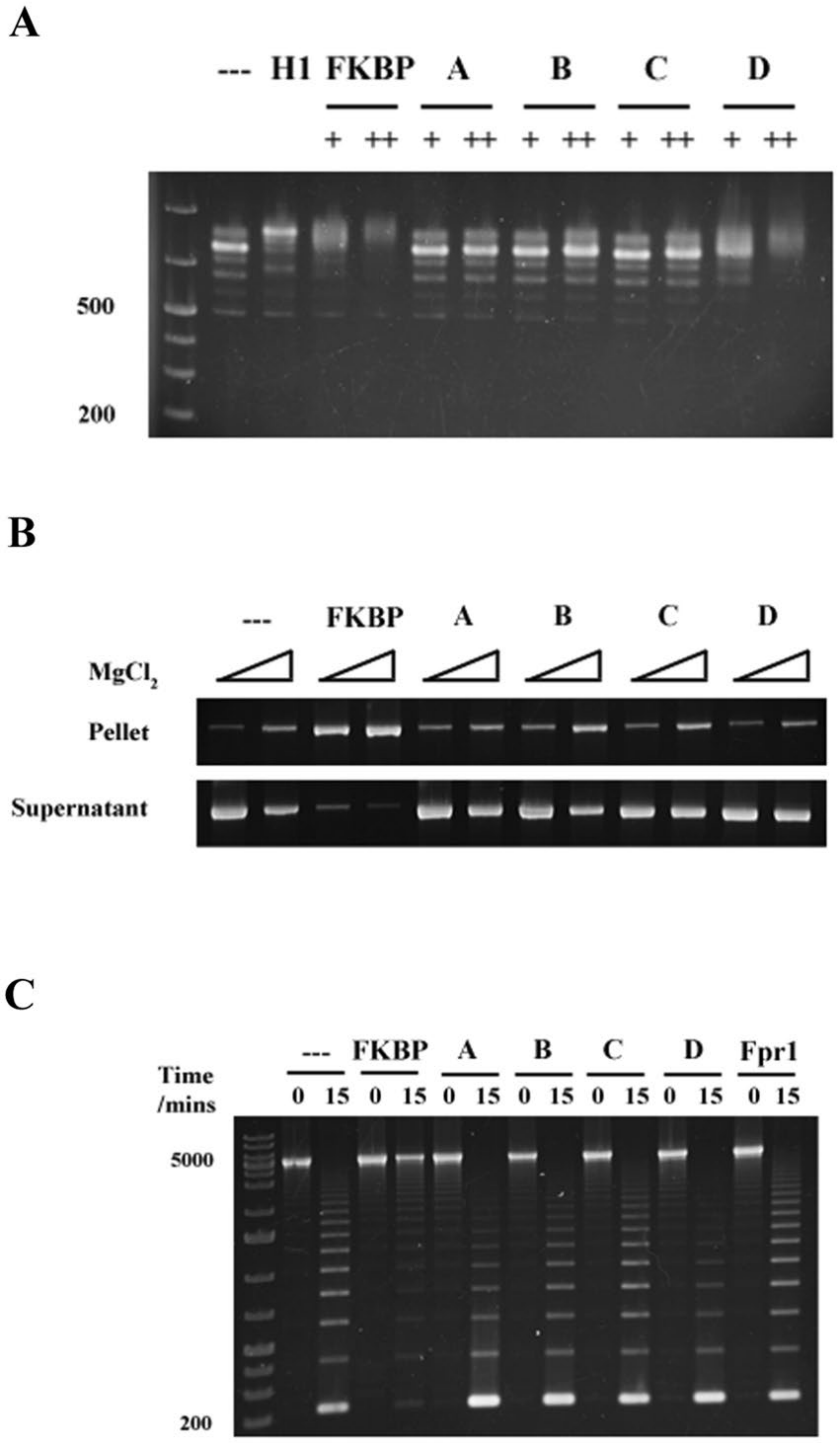
The basic patches of the Fpr4 FKBP are required for chromatin interaction. (**A**) Basic patches are required for FKBP binding to dinucleosomes. Dinucleosomes were mixed with increasing concentrations of histone H1, Fpr4 FKBP, or basic patch mutants A-D proteins and subjected to EMSAs as in Figs 1 and 2. (**B**) Basic patches are required for FKBP promotion of magnesium-dependent chromatin fibre self-association. Recombinant Fpr4 FKBP or basic patch mutants were mixed with 25mer nucleosome array in the presence of 0.25 and 0.5 mM MgCl_2_ and centrifuged. Chromatin in the supernatant and pellet fraction were visualized by agarose gel electrophoresis with ethidium bromide staining. Full-length images of this cropped gel are available in the Supplementary Information File. (**C**) Basic surface patches are required for linker region interaction. Recombinant Fpr1, Fpr4 FKBP, or basic patch mutants were mixed with 25 mer nucleosome array, and arrays digested with AvaI restriction enzyme for 0 or 15 minutes. DNA was purified and subjected to agarose gel electrophoresis and ethidium bromide staining.

To examine if these basic patches facilitate chromatin binding we generated four neutralizing surface charge mutants that revert an Fpr4 basic patch, indicated in leftmost panel of Fig. 4C, to the corresponding Fpr1 amino acid sequence (Mutant A, Green: K280A, K282A, K284A, R295I, K299D; Mutant B, Magenta: K316T, K318E, K321Q; Mutant C, Gold: R312H, K325S, K330S) and Mutant D, Cyan: K305T, R308L, K335N, K392N). Each of these proteins, and Fpr1, were found to be catalytically active, and thus folded, using a H3 Pro30 peptide in chymotrypsin-coupled prolyl isomerase assay^6^ (data not shown). These proteins were then tested for their ability to interact with dinucleosomes (Fig. 5A), precipitate chromatin (Fig. 5B), and condense chromatin by AvaI restriction of nucleosome arrays (Fig. 5C). We find that loss of basic character at these positions either largely reduces (Mutant D) or fully disrupts (Mutants A–C) the interaction of the FKBP with dinucleosomes. Furthermore, these mutants are unable to induce nucleosome array self-association (Fig. 5B) and modify chromatin topology, as measured by AvaI access to linker DNA (Fig. 5C). Together, these data show that the distinguishing basic surface features of the Fpr4 FKBP are required for chromatin binding and array self-association *in vitro*. Since these surfaces are generally conserved in NPL-FKBP proteins, they are likely to associate with chromatin in a similar manner.

## Discussion

A hallmark of nucleoplasmin proteins is the presence of a protease-resistant homopentameric core, as well as multiple acidic tracts that contribute to a histone binding surface^21^. We set out to determine the histone binding determinants of budding yeast Fpr4 with specific attention towards the novel A1/B1 loop that splits the NPL domain, and the highly-acidic A2 region that is immediately downstream of the NPL sequence. We found that the highly acidic A2 region, which has a remarkable 34 Glu/Asp residues in a 58-amino acid stretch, is both necessary and sufficient for histone interaction (Fig. 1C,D). By contrast, the A1/B1 loop within the NPL fold does not directly bind histones, but is required for full binding activity of Fpr4, implying that it plays a minor supporting role in histone association. The SANS structure of the Fpr4 NPL lacking the A1/B1 loop has been determined, and the topology of this scaffold suggests that both the A1/B1 and A2 regions are likely to increase the charged surface area of the Fpr4 NPL pentamer^5^. Thus, highly charged sequences outside and within the NPL domain of Fpr4 form the surface that binds to free histones.

Since the NPL domain directly binds to histone H3/H4 tails, we sought to determine if it can recruit Fpr4 to chromatinized histones. Using EMSAs with both mono and di-nucleosomes we confirm that nucleosomal histones are recognized by the NPL domain. This interaction may anchor Fpr4 for chromatin modification (see below) or facilitate nucleosome association in preparation for an eviction event. Surprisingly, our EMSAs uncovered an unexpected nucleosome-binding property of the Fpr4 catalytic domain. While this FKBP fold does not bind to free histones (Fig. 1C,D) or naked DNA (Fig. 1E), it does bind to mono- and dinucleoseomes. Both DNA and protein components of the nucleosome contribute to this association. Linker DNA is the primary determinant as the FKBP domain can bind recombinant 197 bp but not 147 bp mononucleosomes (Fig. 2A). Since the removal of histone tails, reduces, but does not abolish, the binding of the Fpr4 FKBP to dinucleosomes (Fig. 2C) we speculate that histone tails may modulate this interaction. Regardless, it appears that the FKBP domain of Fpr4 is equipped to directly recognize chromatinized DNA.

A comparative analysis of NPL-containing FKBP domains from fungi, insects and plants, shows that these FKBPs consistently differ from non-chromatin proximal FKBPs. Most noticeably, they are enriched in lysines. These residues form four basic patches that are generally conserved (as opposed to specific lysine positions), suggesting that the highly positively charged characteristics of the FKBP domain is an important trait of chaperones in the NPL-FKBP family. To investigate the importance of these differences we compared yeast Fpr1 and Fpr4. These two proteins contain a nearly-superimposable FKBP fold (Fig. 4B), but the number of basic residues in Fpr4 FKBP is much higher than in Fpr1, resulting in dramatically different isoelectric points for these domains. With a set of Fpr4 to Fpr1 chimeric mutants we show that the basic patches are required for nucleosome binding, chromatin fibre self-association, and linker region interaction (Fig. 5). These observations support the idea that several large areas of positive charge on the FKBP surface are needed to mediate interactions with DNA near or between nucleosomes. This interaction would presumably place the catalytic pocket near its chromatin-proximal substrates. It is notable that in addition to histones, a multiplicity of DNA-proximal proteins has been shown to interact with or be regulated by prolyl-isomerases^22, 23^, including FKBPs^24–27^.

The association of an FKBP prolyl isomerase domain with linker regions provides insight into prolyl isomerase targeting and chromatin regulation. First, this observation demonstrates that while additional domains in isomerases are known to direct prolyl isomerases to distinct proteins, the surface features of the catalytic domains may play an equally important role in substrate recognition. This idea has been proposed for the cyclophilin family^28^, and likely extends to FKBPs. Secondly, the targeting of an FKBP to linker DNA is intriguing because features near the entry/exit region of DNA into the nucleosome heavily influence nucleosome structure and stability and the positioning of histone tails in this region dictates nucleosome-nucleosome interactions. Site-directed cross-linking experiments with nucleosome arrays have clarified the contributions of different tails by showing that the H3 tail mediates inter-array nucleosomal interactions^29^ while the histone H4 tail interacts with linker DNA, and neighbouring nucleosomes through an acidic patch in histone H2A^30^. Our previous experiments have identified potential Fpr4 peptidyl-proline targets in the N-terminal tail of histone H3^6, 9^, but the structural consequences of this action have never been probed using a substrate larger than a free decapeptide^9^. Given these roles for histone tails in chromatin organization, and their known isomerization by Fpr4, we speculated the basic features of the FKBP, and potentially those of the adjacent basic region (B2), would position and/or retain the enzyme near nucleosomes to target core histone tails. This presumably could modify the architecture or outputs of chromatin.

We used recombinant nucleosome arrays to test this idea. Through our assays we demonstrate that while the Fpr4 FKBP interacts with and condenses chromatin, this is strictly a consequence of binding between nucleosomes and is not due to FKBP catalytic action. However, it is still important to appreciate that while histone proline isomerization does not drive chromatin self-association in our *in vitro* system it remains possible that FKBP recruitment to linker DNA directs the catalytic activity to a local target. The fact that the FKBP domain binds to the entry/exit region of the nucleosomes, but not free histones or DNA, means that the proximal histone H3 tails remain a good candidate. The precise consequences of this modification remain unclear, but likely involve the dynamics of either histone tail conformation and/or nucleosome structure(s), and potentially their associations with DNA or proteins. Notably these properties cannot be measured on recombinant 601 nucleosome arrays because such arrays, and the nucleosomes within them, are extremely stable. Therefore, it will be important to test the impact of prolyl isomerase action on nucleosome dynamics on weaker-positioned nucleosomal substrates or ideally in *in vivo* contexts. Additionally, it is possible that the isomerization of histone tails may not affect gross chromatin structure but may instead regulate the association of non-histone proteins with chromatin. Previous work linking H3Pro38 to both Set2 trimethylation and gene induction kinetics^6, 31^ supports the idea that histone tail conformation is important for engagement of non-histone proteins. Alternately, the catalytic pocket may simply bind to histones - while the binding affinity of the FKBP domain for histone H3 tail peptides was previously reported to be weak^9^ (mM range) the stable binding of the domain to nucleosomes may promote this interaction.

The NPL-FKBP family of chromatin proteins have a combination of histone chaperone and histone prolyl-isomerase activities. Here we identify that their FKBP domains also have chromatin binding properties that likely contribute to recruitment of the enzyme to its substrate(s). An understanding of how NPL-FKBPs regulate chromatin will require a careful analysis of the contribution of each of domain *in vivo*. This report has mapped the interfaces between the basic FKBP of these proteins and chromatin. As a result, loss of function histone binding mutants lacking the A2 motif, chromatin-binding basic patch mutants of the FKBP domain and catalytically inactive prolyl isomerase mutants^8, 20^ (F323Y) will be essential in dissecting the molecular details of how these nucleosome-building and histone modifying enzymes cooperate to regulate chromatin.

## Methods

### Protein expression and purification

Human histone proteins were expressed and purified as previously described^17^. Fpr4 NPL domain (and deletion constructs), Fpr4 FKBP domain (WT, F323Y surface charge mutants A-D) and Fpr1 were expressed in *E. coli* BL21 (DE3) as N-terminal 6His tag fusion proteins using the pETHis1a expression vector. Expressed proteins were purified with Ni-NTA agarose resin (Qiagen, cat#30210) using lysis buffer (50 mM Tris pH 7.5, 300 mM NaCl, 10 mM Imidazole, 5% glycerol, 1 ug/mL each of leupeptin (Bio Basic, cat#LDJ691), pepstatin (Bio Basic, cat#PDJ694), aprotinin (Bio Basic, cat#AD0153) and 2mM PMSF (Sigma, cat#P7626)), wash buffer (50 mM Tris pH 7.5, 300 mM NaCl, 20 mM imidazole and 5% glycerol) and elution buffer (50 mM Tris pH 7.5, 300 mM NaCl, 250 mM imidazole and 5% glycerol). Elutions containing purified protein were combined and dialyzed overnight at 4C against 1X TBS +5% glycerol. Site-directed mutagenesis to generate catalytic null Fpr4 F323Y was performed as per standard protocols. Histone H1 was purchased from Roche. DNA corresponding to Fpr4 FKBP mutants A-D was synthesized by Genscript. Mutagenesis to generate Fpr4 NL ΔA2 and ΔA1/B1 was performed using a Q5 Site-Directed Mutagenesis Kit (NEB, cat#E0554S) using primers whose sequences are available upon request.

### Preparation of DNA

1 × 197 bp 601 positioning sequence DNA was prepared as previously described^16^. 2 × 197 bp 601 positioning sequence DNA was prepared as follows: pET19 plasmid containing a 2 × 197 bp insert was digested with *EcoRv*, and the insert DNA was enriched through poly (ethylene glycol) precipitation (4% poly (ethylene glycol) 6000, 0.5 M NaCl, 1X TE, 25000 g for 30′ at 4C). This process was repeated in succession using 5% and 6% poly (ethylene glycol) to further remove plasmid backbone DNA. Finally, insert DNA was purified using Sephacryl S-1000 size exclusion resin (GE Healthcare Life Sciences, cat#17-0476-01). 147 bp 601 positioning sequence DNA was prepared from PCR amplification of 197 bp 601 positioning sequence DNA. Nucleosome array DNA was prepared as previously described^17^.

### Histone octamer, mononucleosome, dinucleosome and nucleosome array reconstitution

Histone octamer assembly and purification, and mononucleosome, dinucleosome and chromatin reconstitutions were performed as per previous protocols^16, 17^. Briefly, purified histone octamer was mixed with DNA in RB high buffer (2 M NaCl, 10 mM Tris pH 7.5, 1 mM EDTA, 1mM DTT) using a 1.1:1 octamer: DNA mole ratio, and dialyzed for 30 minutes at 4C. The final [NaCl] was reduced to 10 mM over 24 hours through the addition of RB low buffer (10 mM Tris pH 7.5, 1 mM EDTA, 1 mM DTT). Reconstituted mononucleosomes, dinucleosomes and nucleosome arrays were visualized through 0.7% agarose gel electrophoresis in 0.2X TB.

### Preparation of tailless dinucleosomes

Reconstituted dinucleosomes were treated with agarose-conjugated trypsin (Thermo Scientific Pierce, cat#20230) for 30 minutes at 37C in digestion buffer (10 mM Tris pH 7.5, 1 mM EDTA, 25 mM NaCl) at a ratio of 5 mg of agarose-conjugated trypsin to 1mg of nucleosomes. Trypsin was removed through centrifugation at 3000 rpm for 3 minutes at 4C. Resulting tailless dinucleosomes were confirmed by western blot analysis (anti-H3 Abcam #ab1791) and imaged using LiCor.

### Protein interactions

Protein interaction experiments were performed using recombinant 6His tagged Fpr4 proteins. Briefly, one microgram of 6His tagged Fpr4 proteins was incubated with two micrograms of recombinant H3 or calf thymus bulk histones (Worthington, cat# LS002548) in interaction buffer (20 mM Tris pH 8, 0.2 mM EDTA, 300 mM KCl, 30 mM Imidazole, 0.1% NP40, 20% glycerol, 2 mM PMSF) for 30 minutes at 4 °C. Equilibrated Ni-NTA agarose beads were added and nutated for 2 hours at 4 °C. Following binding, samples were washed 4 times using interaction buffer. After the last wash, the supernatant was removed and Ni-NTA beads were boiled in 1X SDS loading buffer. Samples were subjected to 15% SDS polyacrylamide gel electrophoresis and transferred to nitrocellulose for western blot analysis. Blots were probed with H3 primary antibody (anti-H3 Abcam #ab1791) and imaged using LiCor.

### Electrophoretic Mobility Shift Assays

Two micrograms of reconstituted mononucleosomes or dinucleosomes were incubated with 0.2 micrograms of the indicated recombinant protein in 1X TE +75 mM NaCl for ten minutes at room temperature and resolved through 0.7% agarose gel electrophoresis in 0.2X TB.

### Restriction Enzyme Accessibility Assay

Two micrograms of reconstituted nucleosome arrays were incubated with 0.2 micrograms recombinant protein for 10 minutes at room temperature in 1X NEB4 buffer (New England Biolabs). AvaI restriction enzyme (12U per timepoint) was added and the reaction quenched on ice in 80% PCR cleanup binding buffer. Timepoints were subjected to PCR cleanup (Bio Basic, cat#BS664) and visualized via 0.7% agarose gel electrophoresis in 0.2X TB.

### Magnesium Dependent Chromatin Self-Association Assay

Two micrograms of reconstituted nucleosome arrays were incubated with 0.2 micrograms recombinant protein for 10 minutes at room temperature in 50 mM Tris pH 7.5 and 75 mM NaCl, followed by addition of magnesium chloride to a final concentration of 0.25 mM, 0.5 mM or 1 mM MgCl_2_. Samples were centrifuged at 21000 g for 30 minutes at 4C, and pellets and supernatants were separated. Each fraction was subjected to PCR cleanup (Biobasic, cat#BS664) and visualized via 0.7% agarose gel electrophoresis in 0.2X TB.

## Electronic supplementary material

Supplementary Information File
