# Supplementary material for: Basic surface features of nuclear FKBPs facilitate chromatin binding
Source: Sci Rep. 2017 Jun 19;7:3795. doi: 10.1038/s41598-017-04194-7 (PMC5476585; doi:10.1038/s41598-017-04194-7)

Supplementary Information File

Full-length gels and blots for:

Figures 1C, D

Figure 2B

Figure 3A, B

Figure 5B

Basic surface features of nuclear FKBPs facilitate chromatin binding

Andrew Leung[1], Francy-Pesek Jardim[1], Neda Savic[1], Yoan R. Monneau[2], Rodrigo González-Romero[3], Geoff Gudavicius[1], Jose M Eirin-Lopez[3], Till Bartke[4,5], Cameron D. Mackereth[2], Juan Ausió[1], and Christopher J Nelson[1*]

Figure 1C

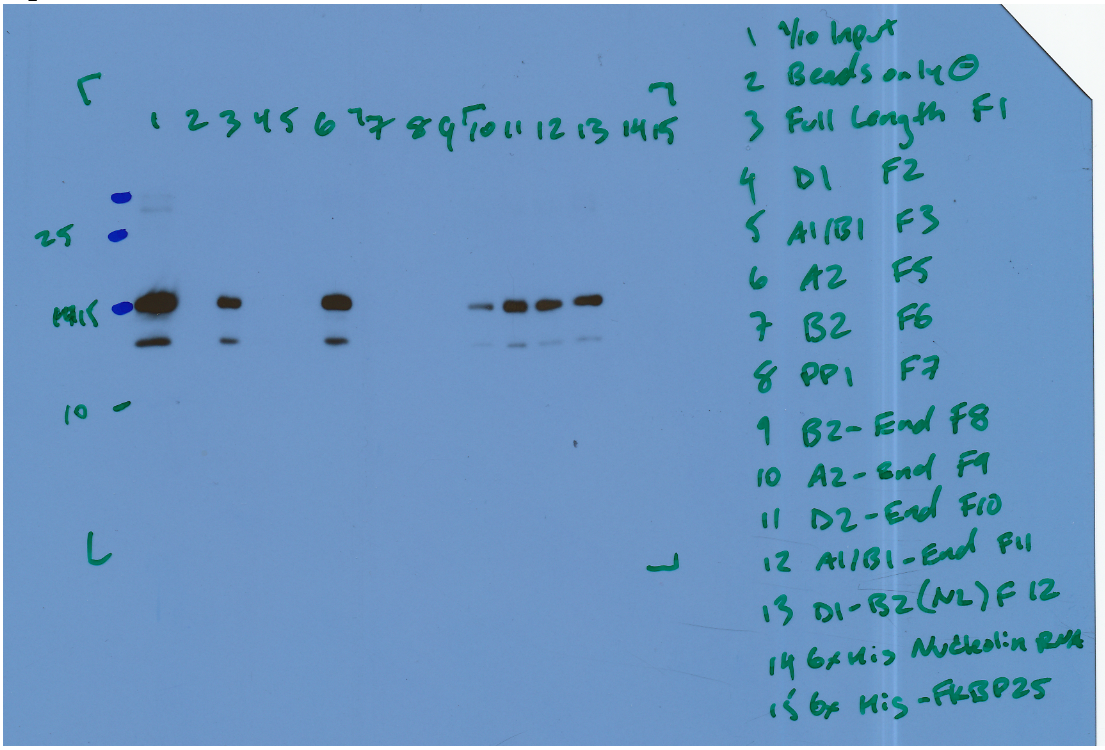

Figure 1D

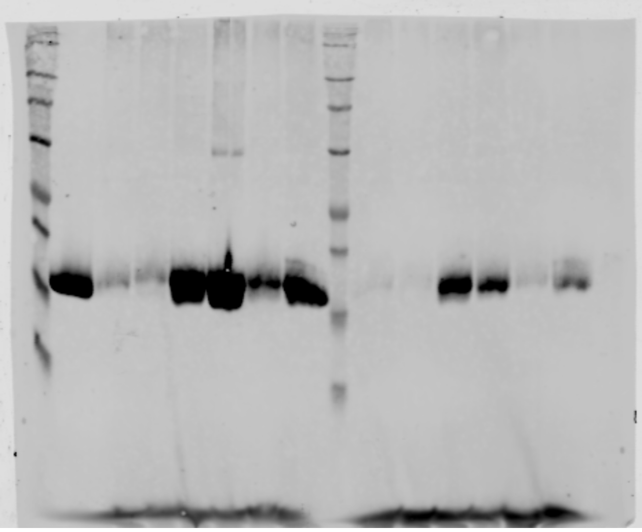

Figure 2B

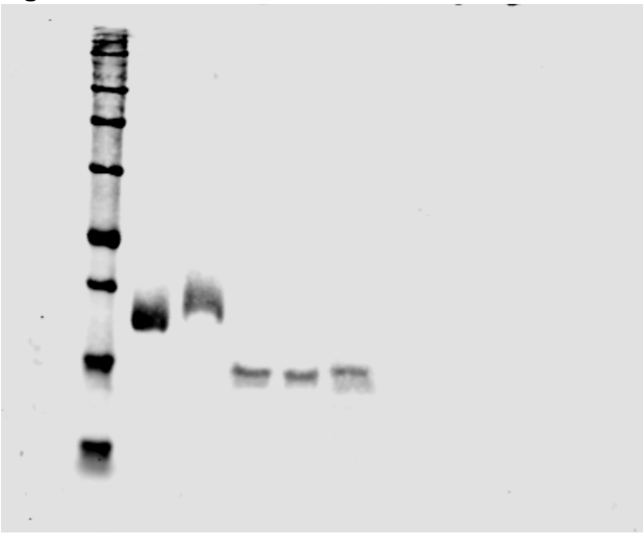

Figure 3A

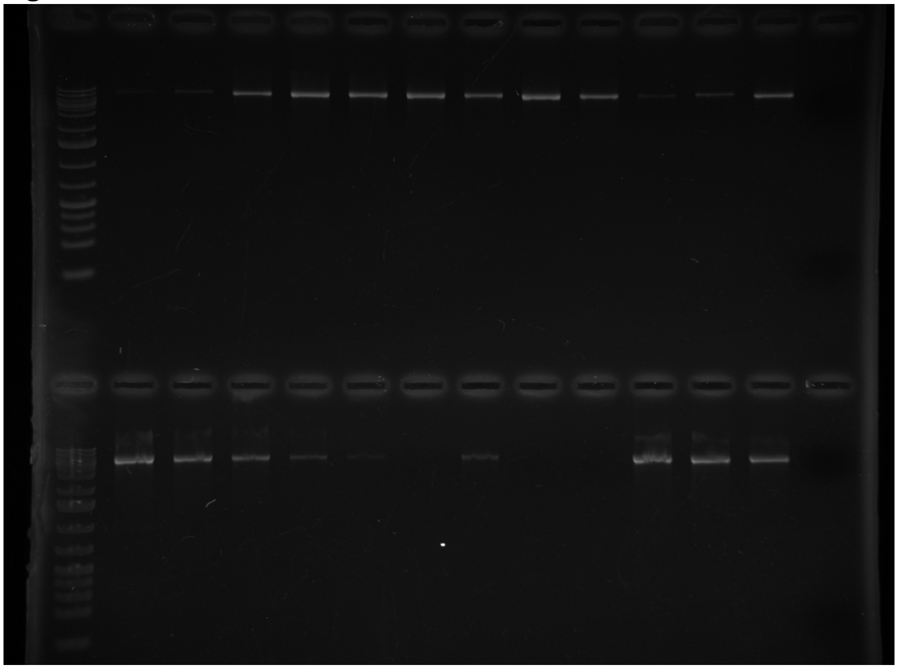

Figure 3B

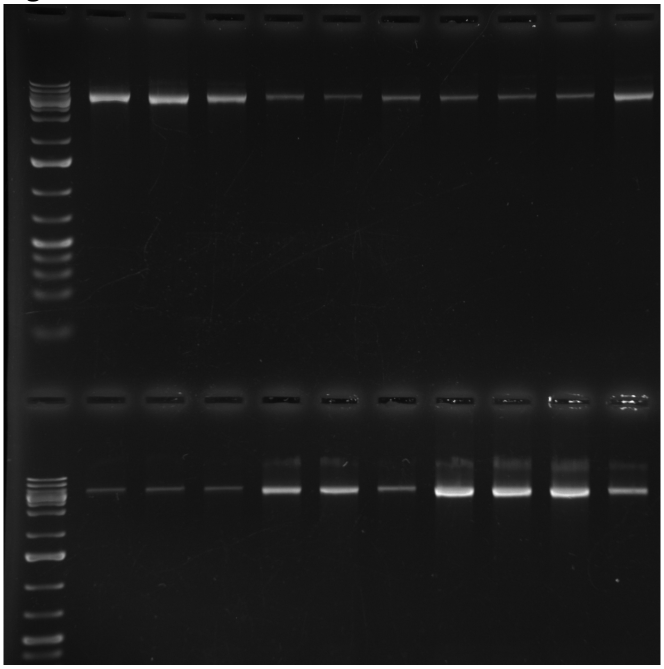

Figure 5B

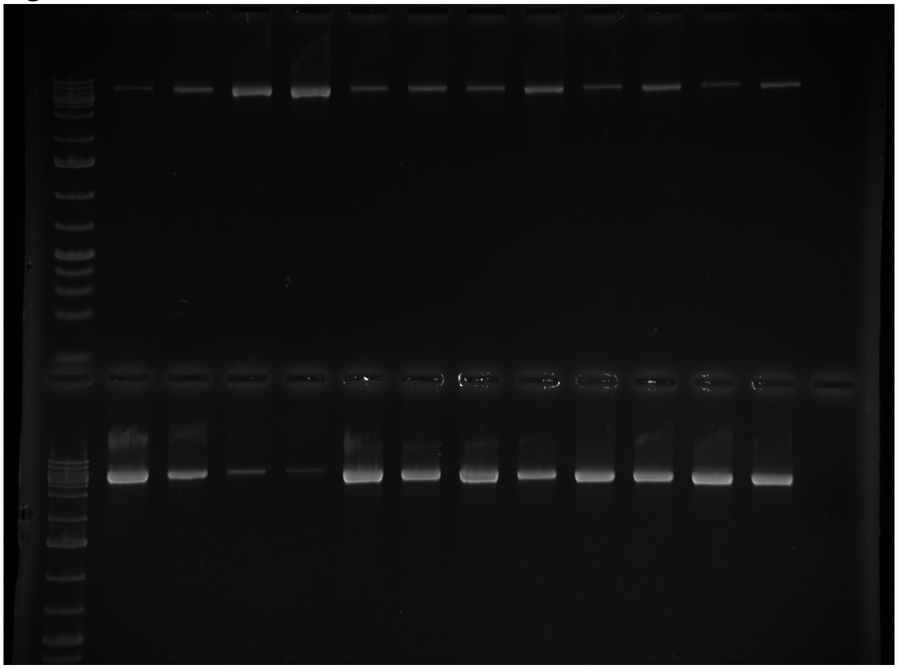

Supplement: Supplementary file 1 — Supplementary Information File [file 41598_2017_4194_MOESM1_ESM.pdf]
